# Distinct Bone Marrow Metabolic Profiles at Relapse Compared with Initial Diagnosis in Pediatric Acute Lymphoblastic Leukemia—An LC-MS Metabolomics Study

**DOI:** 10.3390/cancers18183064

**Published:** 2026-09-21

**Authors:** Sivamoke Dissook, Romteera Kittichaiworakul, Patcharawadee Thongkumkoon, Pitiporn Noisagul, Kanda Fanhchaksai, Lalita Sathitsamitphong, Chane Choed-Amphai, Pimlak Charoenkwan, Parunya Chaiyawat, Apiwat Sangphukieo, Rungrote Natesirinilkul

**Affiliations:** 1 Department of Biochemistry, Faculty of Medicine, Chiang Mai University, Chiang Mai 50200, Thailand; 2 Center of Multidisciplinary Technology for Advanced Medicine (CMUTEAM), Faculty of Medicine, Chiang Mai University, Chiang Mai 50200, Thailand; 3 Thalassemia and Hematology Center, Faculty of Medicine, Chiang Mai University, Chiang Mai 50200, Thailand; 4 Division of Hematology and Oncology, Department of Pediatrics, Faculty of Medicine, Chiang Mai University, Chiang Mai 50200, Thailand

**Keywords:** metabolomics, acute lymphoblastic leukemia, relapse, children, bone marrow, LC-MS

## Abstract

Background/Objectives: Relapse remains the leading cause of mortality in pediatric acute lymphoblastic leukemia (ALL), underscoring the need for novel biomarkers and therapies. The objective of this study is to identify distinct metabolomes associated with relapsed ALL, using bone marrow aspirated at the relapse episode itself, a specimen that is rarely banked and whose metabolome has not previously been reported. Method: This study utilized untargeted liquid chromatography–mass spectrometry (LC-MS) metabolomics on bone marrow aspirates from 21 children with B-lineage ALL. Aliquots from the 12 non-relapsed patients were collected at initial diagnosis and those from the 9 relapsed patients at relapse, so group and disease phase are collinear by design. Results: The resulting metabolic profile clearly distinguished bone marrow sampled at relapse from bone marrow sampled at initial diagnosis. Partial least-squares discriminant analysis separated the two groups, completely in positive ion mode and with 18 of 21 samples correctly classified in negative ion mode (1000-permutation test, *p* = 0.001 in both modes), and 31 of 92 metabolites in positive ion mode and 3 of 44 in negative ion mode differed after Benjamini–Hochberg correction (q < 0.05), of which 26 and 3 respectively also exceeded the fold-change threshold of |log2FC| > 1. The relapse-associated differences involved carnitine, amino acid and lipid metabolites. O-acetylcarnitine was elevated, which is compatible with increased fatty acid oxidation; dipeptides were higher but not significantly (q = 0.075); L-allo-threonine was elevated, which is compatible with increased flux through the serine–glycine one-carbon pathway; adenosine monophosphate (AMP) was decreased, although the abundance of AMP alone does not provide the AMP: adenosine triphosphate (ATP) ratio and cannot establish the activity of AMP-activated protein kinase (AMPK) or of mammalian target of rapamycin complex 1 (mTORC1); and individual lipid species differed in both directions. These are hypotheses generated by the observed differences in metabolite abundance and are not mechanisms demonstrated by this study. Conclusions: This exploratory comparison describes the metabolite composition of bone marrow at relapse and identifies fatty acid oxidation, the serine–glycine one-carbon pathway and ether-lipid metabolism as priorities for future investigation rather than as established therapeutic dependencies. Because the relapsed specimens were obtained at relapse, the signature describes relapsed disease and is not evidence that relapse can be predicted at diagnosis.

## Simple Summary

Bone marrow taken from children at the time of an acute lymphoblastic leukemia (ALL) relapse differs in its metabolite composition from bone marrow taken from other children at initial diagnosis. The differences involve fatty acid, amino acid and lipid metabolites and suggest how relapsed disease may be sustained, although this study measured the abundance of metabolites and cannot establish the activity of the pathways that produce them. These exploratory findings identify metabolic nodes that merit investigation in future work; the study does not show that any of them is a target for treatment. Marrow is rarely collected and stored at the moment a child relapses, so this comparison has not been made before. The bone marrow of the relapsed children was sampled at the time of relapse and that of the non-relapsed children at diagnosis, so this work describes the metabolism of relapsed disease rather than a test that could predict relapse in advance.

## 1. Introduction

Acute lymphoblastic leukemia (ALL) is the most prevalent childhood malignancy, characterized by clonal proliferation of lymphoid precursors [1]. Despite overall survival rates exceeding 80–85% with contemporary therapies, 20–25% of patients eventually relapse, which carries a poor prognosis [2]. Early identification of patients at risk of relapse is therefore crucial. Currently, the presence of measurable residual disease (MRD) at the end of induction is the strongest predictor of relapse in pediatric ALL. However, MRD detection requires specific molecular or flow cytometric markers of leukemia cells [3,4]. There is growing interest in complementary approaches, such as omics technologies, to uncover systemic biomarkers of relapse risk and deepen our understanding of pediatric ALL biology.

Metabolomics, the comprehensive profiling of small-molecule metabolites in biological samples, has emerged as a powerful tool to characterize the biochemical state of cancer patients [5]. In hematological malignancies including ALL, metabolomic and lipidomic analyses have provided insights into disease associated metabolic reprogramming [6]. For example, prior studies have shown that ALL patients exhibit distinct serum metabolite and lipid patterns compared to healthy controls. Alterations in central carbon metabolism, amino acid utilization, and lipid pathways have been linked to treatment response in ALL [7,8]. Schraw et al. [9] demonstrated that metabolic profiles in diagnostic bone marrow could distinguish patients with MRD-positive disease (who are at high relapse risk) from MRD negative patients, highlighting pathways such as glycolysis/tricarboxylic acid (TCA) cycle and amino acid metabolism in early treatment response. Moreover, plasma metabolomics has been used to identify diagnostic and prognostic biomarkers in ALL; for instance, Bai et al. observed significant differences in metabolites (including multiple lysophospholipids) between ALL patients at diagnosis, those in remission, and healthy children [10]. These findings underscore that ALL is accompanied by systemic metabolic perturbations that might correlate with outcomes. Both of these studies, and the pediatric ALL metabolomics literature more generally, examined specimens obtained at initial diagnosis or during first-line treatment. To our knowledge, the bone marrow metabolome of children whose disease has relapsed, measured in marrow aspirated at the relapse episode itself, has not previously been reported. Such specimens are scarce: relapse follows in 20 to 25 per cent of children treated for ALL [2], and only a fraction of those episodes yields marrow that is both aspirated and archived for research.

Lipids and fatty acid metabolism are of particular interest in leukemia pathogenesis. Leukemic cells rely on membrane synthesis and energy production, often displaying dysregulated lipid profiles [11,12]. Glycerophospholipids (such as phosphatidylcholines and phosphatidylethanolamines) and sphingolipids are frequently altered in leukemia patients. For example, a recent metabolomic study in acute leukemia reported widespread downregulation of phosphatidylethanolamines and lysophosphatidylethanolamines in patient plasma relative to healthy controls [13,14]. Sphingolipid metabolism also plays a role in leukemogenesis and drug resistance; certain sphingolipids act as bioactive mediators of cell fate. Notably, sphinganine (dihydrosphingosine), a ceramide precursor, has been identified as a pro-apoptotic sphingolipid, whereas glucosylceramide promotes proliferation [15,16]. In chronic lymphocytic leukemia (CLL), high circulating glucosylceramide correlates with aggressive disease, while higher sphinganine levels associate with longer treatment-free survival. These observations suggest that a balance in sphingolipid species can influence leukemia progression [17,18].

Another aspect of leukemia metabolism is amino acid utilization and its byproducts. ALL cells have increased demand for glutamine and branched-chain amino acids (BCAAs) like leucine, to fuel proliferation and macromolecule synthesis [19,20]. This heightened consumption can lead to accumulation of intermediary metabolites such as dipeptides. For instance, leucylproline (a dipeptide of leucine and proline) was found to be elevated in the plasma of acute leukemia patients, reflecting active BCAA metabolism by leukemic cells. Such dipeptide signatures highlight how leukemia perturbs normal protein turnover and nitrogen metabolism [21,22].

Carnitine and fatty acid oxidation pathways also undergo changes in ALL. Carnitines serve as essential shuttles for fatty acid transport into mitochondria for β-oxidation and are important for tumor metabolic flexibility [23,24]. Previous studies have documented reduced levels of certain acyl-carnitines in ALL patients. Morad et al. [25] observed a significant decrease in O-acetylcarnitine in the plasma of ALL and AML patients compared to controls. It has been suggested that leukemia or its treatment may induce systemic carnitine deficiencies; e.g., chemotherapy can impair carnitine absorption and biosynthesis. Consequently, disruptions in carnitine and fatty acid metabolites could reflect both disease-driven metabolic reprogramming and therapy-related effects [26].

In this study, we employed an untargeted liquid chromatography–mass spectrometry (LC-MS)-based metabolomics approach to compare bone marrow sampled at a relapse episode with bone marrow sampled at initial diagnosis from patients who went on to remain in remission. Our objective was to determine whether their metabolic profiles differ significantly and to identify key metabolites or pathways characterizing each group. We hypothesized that the bone marrow metabolome of relapsed ALL differs from that of newly diagnosed ALL. No non-leukemic comparison group was included, so the study addresses relapse-associated differences within ALL rather than differences between ALL and non-malignant marrow. By integrating multivariate statistical analyses and metabolite identification, we provide a comprehensive overview of the metabolic alterations associated with ALL and its outcomes. This knowledge may nominate biochemical pathways for future investigation in pediatric ALL, and provides a reference profile against which diagnosis-time specimens would need to be compared in order to test whether any part of it is detectable before relapse occurs.

## 2. Materials and Methods

### 2.1. Ethical Approval

This study was conducted following the principles of the Declaration of Helsinki. The research protocol was reviewed and approved by the Ethics Committee of the Faculty of Medicine, Chiang Mai University, Thailand (study number: PED-2566-0638). All bone marrow specimens were obtained from the Biospecimen Banking at the Division of Hematology, Department of Internal Medicine, Faculty of Medicine, Chiang Mai University, Thailand. These were archived, de-identified specimens for which patients had previously provided broad informed consent for future research use (study number: PAT-2562-06932).

### 2.2. Study Populations

After obtaining ethical approval, we retrospectively identified 21 pediatric patients diagnosed and treated at Maharaj Nakorn Chiang Mai Hospital, Faculty of Medicine, Chiang Mai University, whose archived bone marrow cell pellets were available in the institutional biobank. The diagnosis of ALL was established by standard criteria, comprising morphological examination of bone marrow aspirates, immunophenotyping by flow cytometry and cytogenetic analysis by chromosome study. All 21 patients had B-lineage ALL; no T-lineage case was included. The design is a retrospective case-control comparison within ALL. The relapsed group (R group, *n* = 9) comprises every eligible relapsed case held in the biobank over the study period rather than a sample of them, because relapse is uncommon; the non-relapsed comparison group (N group, *n* = 12) was drawn from the same biobank over the same period and is defined as patients who remained relapse-free for at least two years after completing chemotherapy; the follow-up actually achieved in that group is considerably longer, with a median of 5.2 years (range 3.0 to 7.3 years). The cohort is therefore deliberately enriched for relapse and its group proportions do not represent the incidence of relapse in this population. Bone marrow samples were excluded if the archived specimen was insufficient for all investigations. Sampling timepoint: This is the single most important feature of the design and is stated explicitly. Every aliquot from the non-relapsed group was collected at initial diagnosis. Every aliquot from the relapsed group was collected at a relapse episode: at the first relapse in six patients, at the second relapse in two and at the fourth relapse in one. No relapsed patient contributes a diagnosis-time specimen. Group membership and sampling timepoint are therefore completely collinear by construction, and the comparison reported here is between newly diagnosed and relapsed disease rather than a comparison made at a common timepoint. All bone marrow samples were processed using a standardized protocol. Cell pellets were aliquoted into cryovials and stored at −80 degrees C in the Biospecimen Banking at the Division of Hematology, Department of Internal Medicine, Faculty of Medicine, Chiang Mai University, Thailand.

### 2.3. Metabolite Extraction

The extraction protocol was adapted from the method described by Andresen et al. [27]. The process began with the lysis of 2 × 10^7^ cells in 1 mL of isopropanol, achieved by vortexing for 5 min and sonicating on ice for 10 min. For protein precipitation, the samples were incubated at −20 °C for 60 min, after which cellular debris was pelleted by centrifugation at 16,000× *g* and 4 °C for 10 min.

The metabolite-containing supernatant was carefully transferred and filtered through a 0.4 µm syringe filter. Solvent was then removed in a two-step process: samples were first concentrated to approximately one-quarter of their initial volume in a SpeedVac concentrator (Thermo Fisher Scientific, Waltham, MA, USA) at 25 °C, followed by complete evaporation to dryness under a stream of nitrogen. The final dried extracts were stored at −80 °C pending subsequent analysis.

### 2.4. Liquid Chromatography-Mass Spectrometer (LC-MS) Analysis

Metabolic profiling was conducted using a high-resolution mass spectrometry platform. Specifically, an ultra-high-performance liquid chromatography (UHPLC) system coupled to a quadrupole time-of-flight (QTOF) mass spectrometer was employed. Chromatographic separation was achieved on a reverse-phase C18 column (100 mm × 2.1 mm, 1.7 μm particle size) maintained at 40 °C. The mobile phase consisted of solvent A (water containing 0.1% formic acid) and solvent B (acetonitrile with 0.1% formic acid). A linear gradient elution was applied, starting at 5% B and increasing to 95% B over 30 min, held at 95% B for 5 min, and then returned to 5% B for column re-equilibration. The flow rate was set at 0.3 mL/min, with an injection volume of 5 μL per sample.

Mass spectrometric detection was performed using electrospray ionization (ESI), with each sample analyzed in both positive (ESI^+^) and negative (ESI^−^) ionization modes to enhance metabolite coverage. Full-scan MS data were acquired over a mass-to-charge (*m*/*z*) range of 50–1200. The instrument was calibrated prior to analysis, and continuous mass accuracy correction was achieved using two reference ions in each mode. To ensure data quality and system stability, pooled quality control (QC) samples were injected at regular intervals (every 5–10 injections) for monitoring signal consistency and enabling correction for potential instrumental drift.

### 2.5. Data Processing

Raw LC–MS data were converted to an open format (e.g., mzML) and processed using MS-DIAL version 5.3 for feature detection, spectral deconvolution, retention time alignment, and metabolite annotation. Peak detection parameters, including the signal-to-noise ratio (S/N) and minimum amplitude threshold, were optimized empirically (e.g., S/N ≥ 3). Features detected in blank samples were excluded following deconvolution. Retention time correction was performed using internal standards and pooled QC samples to ensure consistency across runs. Positive and negative ionization mode datasets were analyzed independently.

Metabolite annotation was conducted by matching accurate mass and MS/MS spectra against publicly available libraries, including the Human Metabolome Database (HMDB) and LipidBlast, integrated within MS-DIAL. Putative identifications were assigned based on spectral similarity, mass accuracy, and retention time (when available), and categorized by confidence level.

The resulting data matrices were exported for downstream statistical analysis. Prior to analysis, features with high missingness (>20%) or poor reproducibility in QCs were removed. Remaining missing or zero values were replaced by one-fifth of the smallest positive value recorded for that feature, which is the default imputation of MetaboAnalyst. The complete annotated matrices for both ionization modes and all 21 patients are provided in Table S4. Data were then log10-transformed to reduce heteroscedasticity and auto-scaled, that is, each metabolite was mean-centered and divided by its standard deviation so that all metabolites contributed equally to the multivariate models.

### 2.6. Statistical Analysis

Processed data were analyzed using MetaboAnalyst 6.0 and custom scripts in R. Unsupervised principal component analysis (PCA) was applied both to the pooled quality control injections, to confirm analytical stability, and to the 21 patient specimens, to visualize natural clustering and to detect outliers; both results are reported. Supervised partial least-squares discrimination analysis (PLS-DA) was performed to enhance group separation and identify the metabolites most responsible for class discrimination (using variable importance in projection, VIP scores). The PLS-DA model was fitted with two components and its predictive performance was assessed by leave-one-out cross-validation; class-label permutation testing with 1000 permutations was used to confirm that the observed separation exceeded chance. Model fit (R2Y) and cross-validated predictive ability (Q2) are reported for each mode. Univariate comparisons between the relapsed and non-relapsed groups were made with Welch’s two-sample *t*-test on the log10-transformed data, and the resulting *p*-values were corrected for multiple testing across all annotated metabolites using the Benjamini–Hochberg false discovery rate (FDR). A non-parametric Mann–Whitney U test was run in parallel and gave closely concordant results (28 of the 31 significant positive-mode metabolites and all three significant negative-mode metabolites were significant under both tests). Metabolites were considered significantly altered when the FDR-adjusted *p*-value (q) was below 0.05 and the absolute log2 fold change exceeded 1. Fold change was calculated as the ratio of the mean abundance in the relapsed group to that in the non-relapsed group, so that positive values denote higher abundance in relapse. All analyses were carried out in R version 4.3.3, except the sensitivity analysis described below, which was carried out in Python 3.11 and reproduces the R results on every deterministic quantity; the analysis script and the input matrices are provided in the Supplementary Materials so that every figure in this paper can be regenerated from the raw data. As a sensitivity analysis, the complete workflow, from replacement of zero values through logarithmic transformation, autoscaling, Welch’s *t*-tests with Benjamini–Hochberg correction and PLS-DA, was repeated after excluding the three patients whose relapse was confined to the central nervous system. To distinguish a loss of statistical power from an effect specific to those specimens, the same exclusion was applied to all 84 possible subsets of three relapsed patients (Figure S3, Table S5).

## 3. Results

This study analyzed the metabolic profiles of bone marrow aspirates from 21 pediatric patients with B-lineage ALL, comprising 12 non-relapsed and 9 relapsed cases. In the non-relapsed group, sampled at initial diagnosis, the mean age at diagnosis was 4.2 years (median 3.6; range 1.6–12.6). In the relapsed group the mean age at diagnosis was 6.5 years (median 6.5; range 3.0–12.2), and because these aliquots were collected at relapse, the mean age at the time of sampling was 10.5 years (median 10.3; range 4.7–16.3). The median time from diagnosis to first relapse was 27 months (range 5–60 months), and the median interval between diagnosis and collection of the analyzed aliquot was 3.4 years (range 0.5–11.6 years). Relapse was isolated to the bone marrow in six patients (66.7%) and to the central nervous system in three (33.3%). Median follow-up from diagnosis in the non-relapsed group was 5.2 years (range 3.0 to 7.3 years; 36 to 88 months), and all twelve patients remained relapse-free and alive at last contact; one patient was censored at 3.7 years on transfer of care to another center. Detailed clinical and demographic data are summarized in Table 1, and per-patient clinical and metabolite data are provided in Supplementary Tables S1 and S2, with the annotation key for each reported metabolite indicated in Table S3.

**Figure 1:**
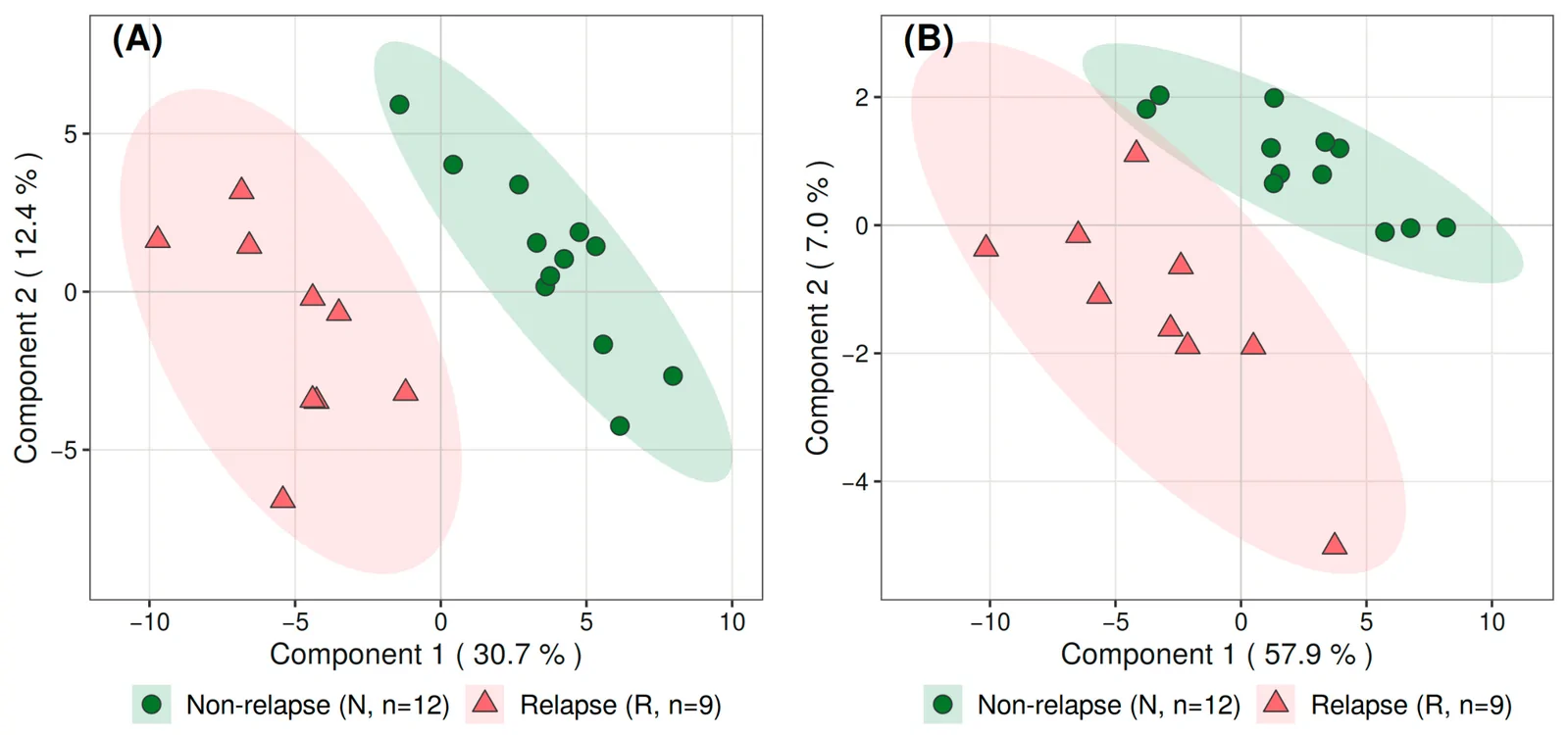
Supervised modeling separates marrow sampled at initial diagnosis from marrow sampled at relapse. The scores plot from the partial least-squares discriminant analysis (PLS-DA) model demonstrates a separation between specimens taken at initial diagnosis in the non-relapsed group (N, green circles) and specimens taken at relapse in the relapsed group (R, red triangles), shown with 95% confidence ellipses; model statistics are given in the Results. (**A**) positive ion mode and (**B**) negative ion mode.

Metabolomic analyses were performed using LC-MS. The analytical stability of the platform was first confirmed by the tight clustering of QC samples in a PCA model. Unsupervised PCA of the 21 patient specimens separated the two sampling stages along the first principal component in both ionization modes (positive mode, PC1 31.6% and PC2 12.8% of the variance, Welch’s *t*-test on PC1 scores *p* < 0.001; negative mode, PC1 58.8% and PC2 8.4%, *p* = 0.019). To investigate metabolic differences between the two sampling stages, a supervised PLS-DA was conducted. The resulting score plots revealed a distinct separation between the non-relapsed (green circles) and relapsed (red triangles) cases for data acquired in both positive (Figure 1A) and negative (Figure 1B) ion modes. This separation indicates that the metabolite composition of marrow sampled at relapse differs from that of marrow sampled at initial diagnosis. The two-component models explained 43.1% (positive mode) and 64.9% (negative mode) of the variance in the metabolite matrix, classified all 21 samples correctly under leave-one-out cross-validation in positive mode (18 of 21 in negative mode), and separated the groups better than chance in 1000 class-label permutations (*p* = 0.001 in both modes; Figure S1). Variable importance in projection scores for both modes is shown in Figure S2. Model fit and predictive ability were R2Y = 0.912 and Q2 = 0.777 in positive mode and R2Y = 0.854 and Q2 = 0.507 in negative mode. Because the model was trained and tested within the same 21 samples, these figures describe the strength of the separation in this cohort rather than predictive accuracy in new patients.

**Figure 2:**
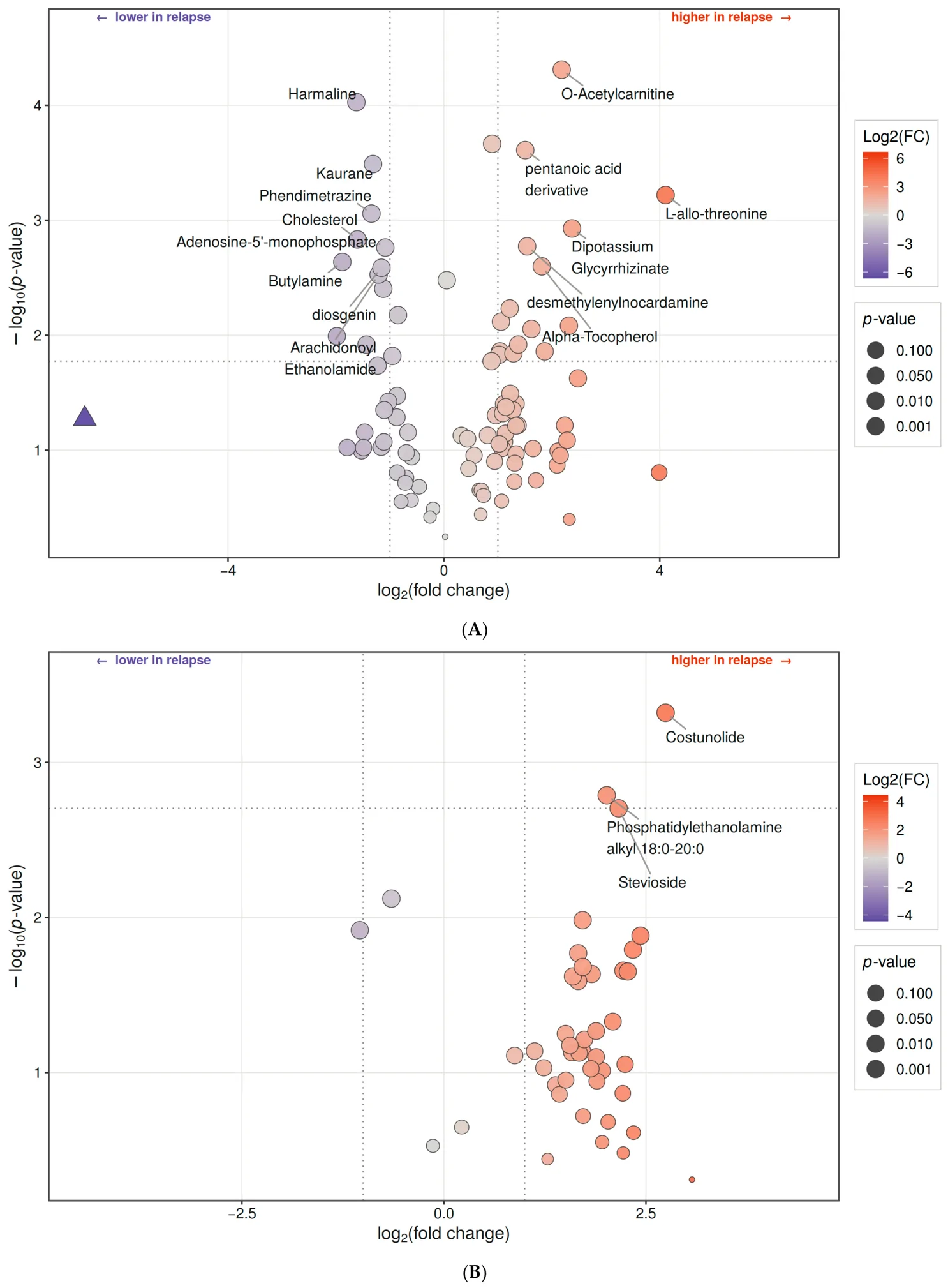
Volcano plots of differentially abundant metabolites in positive (**A**) and negative ion modes (**B**). The plots present statistical significance (−log10 *p*-value) vs. the magnitude of change (log2 fold change) between the relapsed group, sampled at relapse, and the non-relapsed group, sampled at initial diagnosis. Metabolites above the horizontal dotted line (Benjamini–Hochberg q = 0.05) and outside the vertical dotted lines (|log2 fold change| = 1) are considered significantly altered. Fold change is expressed as relapsed/non-relapsed, so that points to the right of the origin are more abundant in the relapsed group and points to the left are less abundant. Point color encodes the log2 fold change and point size the unadjusted *p*-value.

To identify the specific metabolites driving this separation, univariate analysis was performed, and the results were visualized using volcano plots (Figure 2). Metabolites were considered significantly altered if they met the criteria of a Benjamini–Hochberg FDR-adjusted *p*-value (q) below 0.05 together with an absolute log2(fold change) greater than 1.0. Fold change is expressed throughout as relapsed/non-relapsed, so that positive values denote higher abundance in the relapsed group. On this basis, 31 of the 92 annotated metabolites in positive ion mode reached q < 0.05, of which 26 also exceeded the fold-change threshold, whereas only 3 of the 44 metabolites in negative ion mode met both criteria. In positive ion mode (Figure 2A), the metabolites most strongly increased in the relapsed group were L-allo-threonine (log2 fold change +4.11, q = 0.009), dipotassium glycyrrhizinate (+2.37, q = 0.014), O-acetylcarnitine (+2.18, q = 0.004) and alpha-tocopherol (+1.81, q = 0.017), whereas harmaline (−1.62, q = 0.004), cholesterol (−1.60, q = 0.014), kaurane (−1.31, q = 0.006), arachidonoyl ethanolamide (−1.21, q = 0.018) and adenosine-5′-monophosphate (−1.09, q = 0.014) were among those most strongly decreased. In negative ion mode (Figure 2B), the changes were fewer and all in the same direction: costunolide (+2.74, q = 0.021), stevioside (+2.16, q = 0.029) and phosphatidylethanolamine alkyl 18:0–20:0 (+2.01, q = 0.029) were the only metabolites to satisfy both criteria, and all three were more abundant in the relapsed cohort. The diacyl phospholipids PE 40:4 (−0.65) and PG 30:0 (−1.04) were less abundant in relapsed patients but did not survive correction for multiple testing (q = 0.075 for both) and are therefore reported here as trends only.

**Figure 3:**
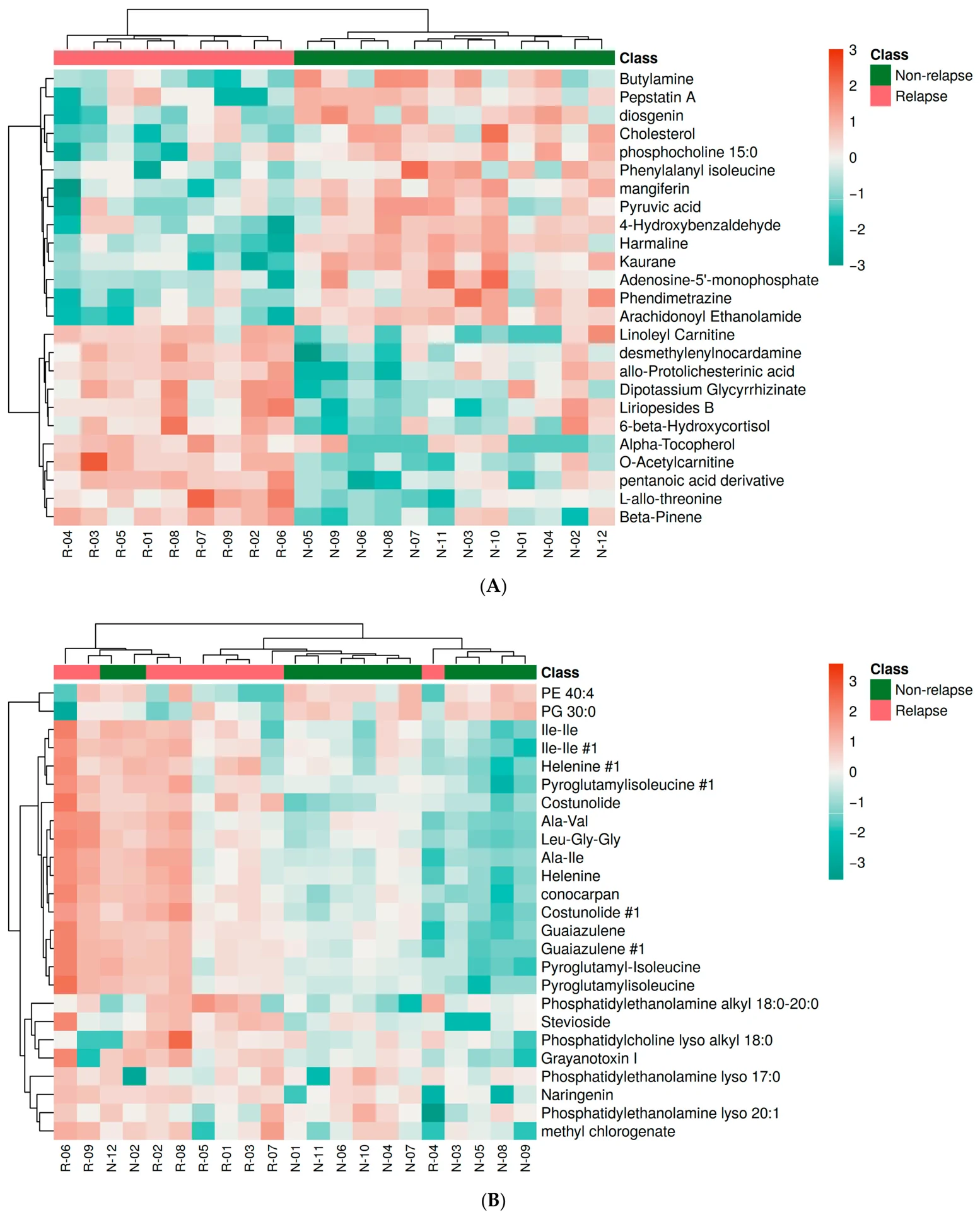
Heatmaps showing the relative abundance of significant metabolites detected in (**A**) positive and (**B**) negative ion modes. Orange indicates high relative abundance; teal indicates low relative abundance. Each column represents an individual patient, sampled at initial diagnosis in the non-relapsed cohort (N) and at relapse in the relapsed cohort (R), and each row represents a single metabolite.

The relative abundance of the most significant metabolites (VIP score > 1 from PLS-DA) was visualized using hierarchical clustering heatmaps to assess patterns across all samples (Figure 3). In positive ion mode (Figure 3A), the heatmap separated the two sampling stages completely on these metabolites, which were themselves selected by VIP score from the supervised model and so are not an independent test of separation. Two clusters of co-regulated metabolites drove this distinction: one group, including O-acetylcarnitine and L-allo-threonine, was upregulated in relapsed patients, while another group, including cholesterol and harmaline, was consistently downregulated. In negative ion mode (Figure 3B), while the unsupervised clustering of patients was less distinct, clear patterns of metabolite abundance were still evident. A specific cluster of metabolites, including costunolide, stevioside and the ether-lipid phosphatidylethanolamine alkyl 18:0–20:0, was consistently more abundant in relapsed patients, whereas the diacyl phospholipids PE 40:4 and PG 30:0 were less abundant; the latter two differences did not reach significance after FDR correction.

**Figure 4:**
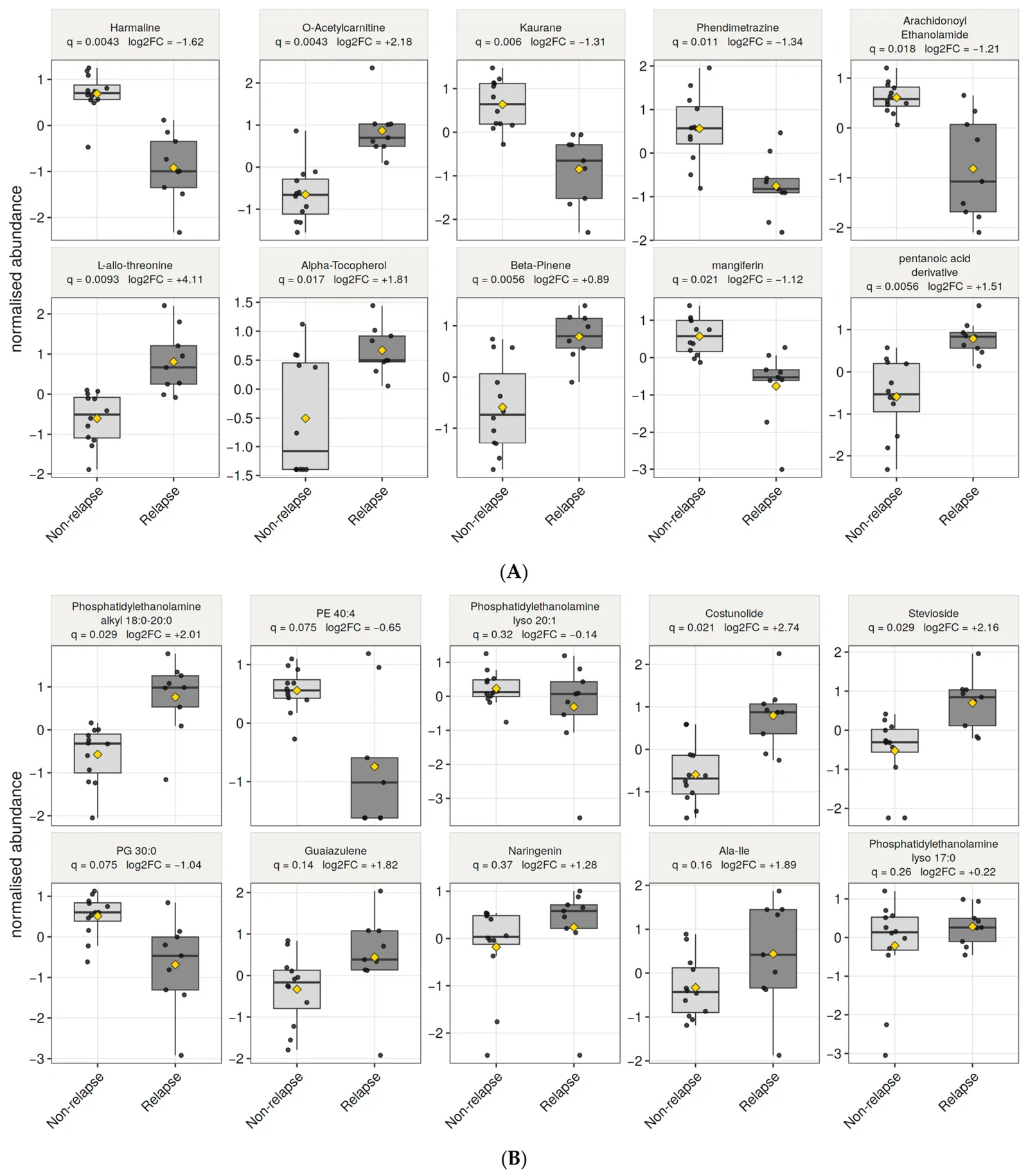
Relative abundance of metabolites in marrow sampled at initial diagnosis and at relapse. Box plots showing the distribution of the 20 most significant metabolites identified from (**A**) positive ion mode (top two rows) and (**B**) negative ion mode (bottom two rows). Each plot compares the relative abundance of a single metabolite between the non-relapsed group, sampled at initial diagnosis, and the relapsed group, sampled at relapse. The box represents the interquartile range, the central line is the median, the yellow diamond is the mean, and individual black dots represent each patient sample. Boxes are shaded light grey for the non-relapsed group and dark grey for the relapsed group.

To confirm these trends, the relative abundances of the top 20 discriminating metabolites from both modes were examined in detail using box plots (Figure 4). In positive mode, a significant increase in O-acetylcarnitine, alpha-tocopherol, L-allo-threonine, pentanoic acid, and dipotassium glycyrrhizinate was confirmed in the relapse group, while adenosine-5′-monophosphate, arachidonoyl ethanolamide, and harmaline were confirmed to be significantly decreased. In negative ion mode, the predominant trend was also upregulation in the relapse group: costunolide, stevioside and phosphatidylethanolamine alkyl (18:0–20:0) were significantly increased (q < 0.05), while gamma-glutamylleucine, leu-gly-gly and taurolithocholic acid showed the same direction of change without reaching significance after FDR correction (q = 0.075). Phosphatidylglycerol (30:0), in contrast, was lower in the relapsed group (log2 fold change −1.04, q = 0.075).

Three of the nine relapsed patients had relapse confined to the central nervous system, although bone marrow was the specimen analyzed in every case. Excluding those three patients and repeating the entire analysis left the direction of change unchanged for all 26 positive-mode and all three negative-mode metabolites that were significant in the full cohort, and the effect sizes were essentially unchanged (Spearman rho = 0.965 across all 92 positive-mode features, with 89 of 92 unchanged in direction, and 44 of 44 in negative mode). Twenty-two of the twenty-six remained nominally significant and eight retained significance under both criteria (q < 0.05 and |log2 fold change| > 1), and the groups remained separated in positive ion mode (R2Y = 0.930, Q2 = 0.748; 16 of 18 specimens correctly classified under leave-one-out cross-validation; permutation *p* = 0.001) and, more weakly, in negative ion mode (R2Y = 0.846, Q2 = 0.329; 14 of 18 correctly classified; permutation *p* = 0.030). Because a fall in the number of significant features could reflect either the removal of those specimens or simply the loss of statistical power, the exclusion was repeated for all 84 possible subsets of three relapsed patients: a median of 13 of the 26 metabolites retained significance across those subsets (range 4 to 21), the subset formed by the three isolated central nervous system relapses lay within that distribution, and the direction of change was preserved for all 26 metabolites in all 84 subsets; the isolated central nervous system subset ranked ninth lowest of the 84 subsets. Which metabolites lose formal significance is reported fully in Table S5 and is stated here because it bears on the interpretation: O-acetylcarnitine remains significant, whereas L-allo-threonine, adenosine-5′-monophosphate, arachidonoyl ethanolamide and the ether-lipid phosphatidylethanolamine alkyl 18:0–20:0 do not, so the reduced cohort supports the existence of a difference between the two sampling stages more strongly than it supports any individual pathway interpretation (Figure S3, Table S5).

## 4. Discussion

This study identified a distinct metabolite profile in bone marrow sampled at relapse, compared with bone marrow sampled at initial diagnosis from children who went on to remain in long-term remission, a difference that is consistent with metabolic reprogramming in the relapsed phenotype. The specimens are the scarce element of this work. Metabolomic studies in pediatric ALL have hitherto profiled marrow obtained at diagnosis [9] or peripheral blood [10], and marrow aspirated at a relapse episode and archived for research is uncommon at any single center; the nine relapse-stage specimens analyzed here are every eligible case banked at this institution over the study period. The observed alterations, spanning fatty acid oxidation, amino acid metabolism, cellular bioenergetics, and lipid composition, are distributed across several pathways rather than confined to one, a pattern compatible with an integrated program of metabolic flexibility, biosynthetic capacity and raised apoptotic threshold, although the present data cannot establish that such a program drives chemoresistance or disease recurrence. The breadth of the altered pathways suggests that relapse is unlikely to involve a single metabolic adaptation. This would be consistent with relapsed cells being not merely faster growing but more adaptable and resilient survivors of intense therapeutic pressure, a concept known as metabolic plasticity that is increasingly recognized in refractory cancers [28].

The observed differences span several compartments, beginning with the structure of the cell membrane. Several lipid species differed between the two groups, the clearest change being the significant enrichment of the ether phospholipid phosphatidylethanolamine alkyl (18:0–20:0) in the relapsed group (log2 fold change +2.01, q = 0.029). Enhanced synthesis of phospholipids is a prerequisite for the rapid membrane biogenesis required to support uncontrolled cell division [29]. Ether lipids in particular are consistently reported to be elevated in aggressive tumors, where they are produced by enzymes such as alkylglyceronephosphate synthase (AGPS) and promote malignant progression, motility and survival, in part by modulating iron uptake and susceptibility to ferroptosis [30]. Their higher abundance in the relapsed cohort is therefore compatible with such a membrane phenotype, although a difference in the abundance of individual lipid species does not by itself establish membrane fortification, inhibition of apoptosis or chemoresistance. The ether-lipid biosynthetic route is identified here as a candidate for future investigation rather than as a demonstrated dependency. The diacyl phospholipids PE (40:4) and PG (30:0) moved in the opposite direction and were less abundant in relapsed patients, but neither change survived correction for multiple testing (q = 0.075 for both), so neither should be over-interpreted in a cohort of this size. The lipid signature is nonetheless consistent with a metabolically flexible and highly energetic state. Alongside these lipid differences, we observed a significant downregulation of arachidonoyl ethanolamide (AEA), an endocannabinoid also known as anandamide. AEA is a potent signaling molecule that induces apoptosis in various cancer cell types, often mediated through the generation of reactive oxygen species (ROS) and the subsequent production of J-series prostaglandins [31]. A lower intracellular concentration of this endogenous pro-apoptotic signal would be compatible with a raised threshold for programmed cell death, a classic hallmark of cancer, although no measure of apoptotic signaling was made in this study. The higher abundance of ether lipids alongside the lower abundance of pro-apoptotic AEA is the pattern that such a coordinated strategy would produce, and it is offered here as a hypothesis for testing.

Amino acid metabolites also differed between the two groups, which would be consistent with a reprogramming of amino acid metabolism to secure nutrients for growth [32], although the present data do not establish that. We observed higher levels of the dipeptides gamma-glutamylleucine and leu-gly-gly, which are typically products of protein breakdown, although neither difference survived correction for multiple testing (q = 0.075 for both). If real, their accumulation would be consistent with enhanced extracellular proteolysis and nutrient scavenging [33,34]. Recent work has shown that cancer cells can thrive in nutrient-poor microenvironments by cooperatively secreting aminopeptidases, such as carnosine dipeptidase 2 (CNDP2), which digest extracellular oligopeptides into free amino acids that can be imported and utilized by the entire tumor population [33]. Whether relapsed ALL cells adopt this “nutrient scavenging” strategy to flourish within the competitive bone marrow niche cannot be determined from dipeptide abundance alone, particularly as the dipeptide differences did not survive correction for multiple testing. The data would also be consistent with relapsed cells rewiring internal amino acid pathways to fuel biosynthetic processes. A key finding was the significant increase in L-allo-threonine, a diastereomer of threonine that can be catabolized by enzymes like serine hydroxymethyltransferase (SHMT) to produce glycine [35]. Glycine, along with serine, is a central hub metabolite that fuels the serine–glycine one-carbon (SGOC) metabolic network [36]. The SGOC pathway is paramount in cancer, providing the one-carbon units necessary for the de novo synthesis of purines and pyrimidines for DNA/RNA replication, the production of the master antioxidant glutathione (GSH) to combat chemotherapy-induced oxidative stress, and the generation of S-adenosylmethionine (SAM) for epigenetic regulation [36]. The elevated L-allo-threonine in relapsed cells is compatible with increased flux into the SGOC pathway, but the abundance of a single metabolite cannot establish flux through a pathway, and this remains a hypothesis to be tested by labeled-substrate or enzymatic measurement. Tryptophan warrants separate comment, because its consumption by indoleamine 2,3-dioxygenase 1 (IDO1) is a well-described immunosuppressive checkpoint: IDO1 depletes local tryptophan, which cytotoxic T cells require to proliferate, and generates kynurenine, which promotes differentiation of immunosuppressive regulatory T cells and can directly induce T-cell apoptosis, and high IDO1 activity is associated with poor prognosis and immune escape in several hematological malignancies [37]. In this cohort, however, tryptophan was slightly higher in the relapsed group (log2 fold change +1.58) and the difference did not reach significance after correction for multiple testing (q = 0.134). Our data therefore provide no support for increased IDO1 flux in relapsed marrow. The question is not settled by this result, because an untargeted measurement of tryptophan alone cannot separate consumption from supply; a targeted assay of the tryptophan-to-kynurenine ratio would be needed to address it directly.

A further difference was found in a metabolite central to cellular energy sensing. We identified a significant decrease in intracellular adenosine-5′-monophosphate (AMP). In a healthy cell, a rising AMP:ATP ratio signals energy distress and activates AMP-activated protein kinase (AMPK), the cell’s master energy sensor and a potent tumor suppressor [38,39]. Lower AMP abundance in relapsed cells would be consistent with a state of perceived energy sufficiency and therefore with reduced activation of AMPK, which normally enforces a metabolic checkpoint that shuts down energy-consuming anabolic processes. That inference is indirect: AMPK responds to the AMP:ATP ratio rather than to AMP abundance alone; ATP was not quantified here, and neither AMPK nor mTORC1 activity was measured. Reduced AMPK activity is known to disinhibit the mammalian target of rapamycin complex 1 (mTORC1) signaling pathway [40,41], which has been described as a driver of poor prognosis and chemoresistance in ALL. The present data are compatible with that axis but do not demonstrate it. No inference about high-risk disease is drawn here, because eight of the nine relapsed patients in this cohort were classified as standard risk by National Cancer Institute criteria at diagnosis. In other systems, hyperactive mTORC1 increases protein synthesis, lipid synthesis and overall cell growth [42]. Such an anabolic state would require a supply of energy and biosynthetic precursors, and O-acetylcarnitine, which was higher in the relapsed group, is one candidate source. O-acetylcarnitine is essential for the carnitine shuttle system, which transports fatty acids into the mitochondria for β-oxidation or fatty acid oxidation (FAO) [28]. An increased reliance on FAO is a hallmark of metabolic plasticity in therapy-resistant cancers, allowing them to generate large quantities of ATP from lipids when glucose is limited [43]. Elevated O-acetylcarnitine is compatible with such an increase but does not establish it, because acylcarnitine abundance reflects the size of a pool rather than the flux through it. Furthermore, O-acetylcarnitine serves as a highly mobile pool of acetyl groups, donating its acetyl moiety to form acetyl-coenzyme A (acetyl-CoA) in the cytoplasm and nucleus. This acetyl-CoA is not only a primary building block for the de novo lipid synthesis promoted by mTORC1 but is also the essential substrate for histone acetyltransferases (HATs). By providing acetyl groups for histone modification, an altered metabolic state could in principle influence the epigenetic landscape [28]. Neither histone acetylation nor gene expression was measured in this study, so this is raised only as a direction for further work.

In summary, the metabolite differences observed in this study are individually modest but point in a consistent direction, and together they generate a testable model of the relapsed ALL clone: one that senses energy sufficiency and thereby permits mTORC1 signaling, diversifies its fuel sources through fatty acid oxidation and amino acid scavenging, increases biosynthetic capacity through the SGOC pathway, and alters membrane lipid composition and apoptotic thresholds [36,43]. Every element of that model is a hypothesis generated by differences in metabolite abundance, and none of them is demonstrated by the present measurements. The downregulation of harmaline, a plant-derived compound with known anti-proliferative activity [44], would be consistent with such a pro-survival state; costunolide, which has comparable activity in other tumor models [45], moved in the opposite direction and was more abundant in relapsed marrow, so no single interpretation accounts for both. The upregulation of taurolithocholic acid (log2 fold change +1.66, q = 0.075) is harder to place. Taurolithocholic acid is an agonist of the membrane bile acid receptor TGR5, whose activation modulates proliferation through AKT, ERK and STAT3 signaling, although the direction of that effect differs between tumor types [46]. Whether the elevation observed here reflects such a signal, or systemic metabolic dysregulation along a gut–liver–marrow axis, cannot be determined from these data; the difference did not survive correction for multiple testing and we offer it only as a hypothesis. A general caveat applies to this whole group of features: harmaline, kaurane, costunolide, stevioside, diosgenin, mangiferin and dipotassium glycyrrhizinate are all plant-derived compounds annotated at MSI level 2 by spectral library matching. Their presence in bone marrow most plausibly reflects diet, herbal or medicinal exposure, or annotation ambiguity rather than endogenous leukemic metabolism, and they were not used to support the mechanistic hypotheses above.

While this study provides a detailed snapshot of the metabolic landscape of relapsed ALL, its limitations must be acknowledged. The most important is structural: because every non-relapsed aliquot was taken at diagnosis and every relapsed aliquot at relapse, group and disease phase are completely collinear. The differences reported here therefore reflect the combined contribution of relapse biology, disease phase and cumulative chemotherapy exposure, and cannot be attributed to any one of them; in particular they are not a diagnosis-time signature and provide no evidence that relapse can be anticipated from a specimen taken at diagnosis. Three of the nine relapsed aliquots came from a later relapse episode, two from a second relapse and one from a fourth, so the relapsed group includes heavily pre-treated disease. The same design also produces an age difference: the groups differ modestly in age at diagnosis (4.2 versus 6.5 years) but substantially in age at sampling (4.2 versus 10.5 years), and the pediatric metabolome changes appreciably across that span. Several clinical variables could not be retrieved, and the reasons differ in a way that matters. The blast percentage of the specific aliquot that was extracted was never measured on these archived specimens; minimal residual disease at day 15 is not part of routine practice at our center and was performed for no patient; and a recurrent fusion gene panel is not reimbursable locally and was likewise not sent for any patient, with Philadelphia chromosome testing at diagnosis restricted to those presenting with hyperleukocytosis. These omissions apply equally to both groups and so do not introduce differential ascertainment, although the absence of aliquot blast percentage means that differences in leukemic cell content between specimens cannot be excluded as a contributor to the profiles reported here. This caveat applies with particular force to the three patients whose relapse was confined to the central nervous system, because bone marrow was the specimen analyzed for every patient. Absence of concurrent marrow involvement in those three was established on the routine diagnostic bone marrow examined at the sampled relapse episode, which showed fewer than 5 per cent blasts on smear, that is, morphological remission of the marrow, against 90 to 98 per cent blasts in the six patients whose relapse was in the bone marrow (Table S1). That examination was performed on the routine diagnostic specimen and not on the archived aliquot: the biobank does not count blasts in the cryovial that is stored, so the leukemic cell content of the three specimens actually extracted for metabolomics remains unknown, and their profiles may derive from marrow that was largely non-leukemic. More generally, the profiles reported here may in part reflect the cellular composition and physiological state of the marrow specimens rather than an intrinsic metabolic program of relapsed leukemia cells. Excluding those three patients did not change the direction of any of the differences reported above (Figure S3, Table S5). End-of-induction minimal residual disease is a different case and we report it explicitly. Flow cytometric MRD is not available at our center, and the only patients with a result are those whose families were able to fund the test privately. Its availability therefore reflects ability to pay rather than clinical indication, and in this cohort it is present for five of the twelve non-relapsed patients and for none of the nine relapsed patients. The variable is completely confounded with group and we have not used it as a covariate. More broadly, the same resource constraint means that socioeconomic circumstances differ across this cohort in ways we could not measure and cannot adjust for; one relapsed patient discontinued treatment for socioeconomic reasons. Whether this contributes to the metabolic differences we describe cannot be determined from these data, and we raise it because it is a limitation that metabolomic studies conducted in resource-constrained settings rarely state. Further limitations follow from the size and provenance of the cohort. Relapse occurs in 20 to 25 per cent of children treated for ALL [2], and the subset of these children for whom a bone marrow specimen was kept in the biobank at the time of relapse is smaller still: the nine relapsed patients analyzed here are every eligible case held in the institutional biobank over the study period, not a sample drawn from a larger pool. Accrual is therefore bounded by the incidence of the event and by what a single center can bank, and no design available to us could have produced a larger series within this period. Twenty-one specimens nonetheless constitute a small series in which many clinical and treatment variables cannot be controlled, and the results are presented as exploratory observations that require confirmation in an independent cohort, ideally one that includes diagnosis-time specimens from patients who subsequently relapse. The adequacy of follow-up in the non-relapsed group can now be stated rather than assumed. Every non-relapsed patient was observed for longer than the median time to first relapse in the comparison group (2.25 years); eight of the twelve were followed beyond 4.5 years and six beyond 5.0 years, intervals by which eight and all nine respectively of the nine observed relapses had occurred. The group is therefore unlikely to contain a substantial number of patients misclassified through short observation. It is not immune to this, however: six of the twelve have not yet been followed beyond the latest first relapse observed here, which occurred at 5.0 years, and one patient in this series relapsed again 11.6 years after diagnosis, so a late event cannot be excluded. A cohort of this size is powered only to detect large effects, and the complete separation achieved by the PLS-DA model should be read as a description of these 21 samples rather than as evidence of predictive accuracy; leave-one-out cross-validation and permutation testing guard against over-fitting but are no substitute for external validation, which this study cannot provide from within itself. We therefore present this work as a pilot and hypothesis-generating study rather than as a validated finding, and we release the complete annotated feature matrices for both ionization modes and all 21 patients, together with the per-patient clinical annotation and the full analysis code, so that these data can be pooled with other cohorts and re-analyzed independently. In a setting where single centers accrue relapsed cases slowly, aggregation across published datasets is the realistic route to validation, and that route requires that the underlying data be published rather than withheld. Future research must focus on functional validation of these hypotheses using in vitro and in vivo models. For instance, inhibiting FAO with an agent such as etomoxir could be tested for its ability to re-sensitize relapsed cells to chemotherapy [47], and a targeted tryptophan-to-kynurenine assay would establish whether IDO1 blockade is worth pursuing in this setting. The decisive next study is one that analyses diagnosis-time specimens from patients who subsequently relapse, which is the only design that can test whether any part of this signature is present before relapse occurs. Finally, unexpected findings like the upregulation of taurolithocholic acid highlight the need to investigate systemic metabolic interactions, such as the gut–marrow axis, which represents a novel and promising frontier in understanding and ultimately preventing relapsed ALL [48].

## 5. Conclusions

In conclusion, this exploratory study found that bone marrow sampled at relapse differs in metabolite composition from bone marrow sampled at initial diagnosis, and that the differences are distributed across lipid, amino acid and energy metabolism rather than confined to a single pathway. The pattern is compatible with a multi-pronged survival strategy, but the study measured the abundance of metabolites only and does not establish that lipid remodeling fortifies membranes, dismantles apoptotic signaling or confers resistance to chemotherapy. Changes in amino acid and dipeptide levels raise the possibility of altered amino acid utilization and nutrient scavenging, although the dipeptide differences did not reach significance after correction for multiple testing. Lower intracellular AMP would be consistent with reduced AMPK activation and consequent mTORC1 signaling, and higher O-acetylcarnitine with an increased reliance on fatty acid oxidation, but neither kinase activity nor pathway flux was measured here, so both remain hypotheses rather than findings. Taken together, these observations identify fatty acid oxidation, the serine–glycine one-carbon pathway, ether-lipid synthesis and AMPK-mTORC1 signaling as priorities for future investigation; this study does not demonstrate that any of them is a therapeutic dependency in relapsed ALL. Because every relapsed specimen in this study was obtained at relapse, these findings characterize the metabolism of relapsed disease and do not establish that the signature is detectable before relapse occurs; testing that will require validation in an independent cohort that includes diagnosis-time specimens from patients who subsequently relapse. We present this cohort as a pilot and, because relapse-stage bone marrow is rarely banked and, to our knowledge, has not previously been profiled in this disease, we publish the complete per-patient metabolite and clinical data so that the community can pool, re-analyze and test these observations rather than await a larger series from one institution.

## Figures and Tables

**Table 1:** Descriptions of the cohort.

	Non-relapsed group (*n* = 12)	
Age at diagnosis (years)	Mean 4.2; median 3.6 (range 1.6–12.6)	
Sex (*n*,%)	Female (7, 58.3%)	Male (5, 41.7%)
NCI risk group (N, %)	Standard risk (10, 83.3%)	High risk (2, 16.7%)
Lineage (*n*, %)	B-ALL (12, 100%)	T-ALL (0, 0%)
Sampling timepoint	At initial diagnosis (12, 100%)	
Follow-up from diagnosis (years)	Median 5.2 (range 3.0–7.3)	
	Relapsed group (*n* = 9)	
Age at diagnosis (years)	Mean 6.5; median 6.5 (range 3.0–12.2)	
Sex (*n*, %)	Female (4, 44.4%)	Male (5, 55.6%)
Relapse site (*n*, %)	Isolated bone marrow (6, 66.7%)	Isolated CNS (3, 33.3%)
Time to first relapse (months)	Median 27 (range 5–60)	
Age at sample collection (years)	Mean 10.5; median 10.3 (range 4.7–16.3)	
Interval, diagnosis to sampling (years)	Median 3.4 (range 0.5–11.6)	
Sampling timepoint	At relapse (9, 100%): first relapse 6, second 2, fourth 1	
Lineage (*n*, %)	B-ALL (9, 100%)	T-ALL (0, 0%)
NCI risk group (*n*, %)	Standard risk (8, 88.9%)	High risk (1, 11.1%)

*n* = number of participants; CNS: central nervous system. Time to first relapse is measured from diagnosis to the first relapse episode. In three patients the analyzed aliquot was taken at a later relapse, as shown in the sampling timepoint row; for those patients the interval from diagnosis to sampling is the longer of the two figures given above.

## Data Availability

All data supporting the findings of this study are provided with the article. The complete annotated metabolite abundance matrices for both ionization modes and all 21 patients, the per-patient clinical annotation, the metabolite annotation key and the full R analysis code with session information, together with the Python code for the sensitivity analysis reported in Figure S3 and Table S5, are supplied in the Supplementary Materials, so that every figure and statistic reported here can be reproduced and the data can be re-used in pooled analyses. Individual-level data are reported in de-identified form, without names, identifiers or dates, in accordance with the biobank consent under which the specimens were held. Raw instrument files are available from the corresponding author on reasonable request.

## Supplementary Materials

The following supporting information can be downloaded at: https://www.mdpi.com/article/10.3390/cancers18183064/s1, Table S1: Per-patient clinical, immunophenotypic and cytogenetic characteristics; Table S2: Per-patient abundance of the discriminating metabolites; Table S3: Metabolite annotation key with MS-DIAL alignment identifiers, fold changes, FDR-adjusted *p* values and annotation confidence; Table S4: (a,b) Complete annotated feature matrices for both ionization modes and all 21 patients; Table S5: (a–d) Per-metabolite results of that sensitivity analysis together with all 84 leave-three-relapsed-patients-out subsets; and the complete R analysis code, input matrices, results tables and session information; and the Python code for the sensitivity analysis; Figure S1: PLS-DA class-label permutation tests for both ionization modes; Figure S2: Variable importance in projection scores for both ionization modes; Figure S3: Sensitivity analysis excluding the three patients whose relapse was confined to the central nervous system.
