# Supplementary material for: Distinct Bone Marrow Metabolic Profiles at Relapse Compared with Initial Diagnosis in Pediatric Acute Lymphoblastic Leukemia—An LC-MS Metabolomics Study
Source: Cancers (Basel). 2026 Sep 21;18(18):3064. doi: 10.3390/cancers18183064 (PMC13605915; doi:10.3390/cancers18183064)
Supplement: Supplementary file 1 [file cancers-18-03064-s001.zip › cancers-4503577-supplementary figures.pdf]

Supplementary Materials

Distinct Bone Marrow Metabolic Profiles at Relapse Compared with Initial Diagnosis in Pediatric Acute Lymphoblastic Leukemia—An LC-MS Metabolomics Study

Contents

Supplementary Figures S1, S2 and S3 are presented in this document with their legends. Supplementary Tables S1–S5 are supplied as a separate Microsoft Excel workbook (cancers-4503577-supplementary Tables.xlsx), one worksheet per table, because their size and structure are unsuitable for a text document. The complete R analysis code, the input matrices, the results tables and the session information are supplied in the accompanying Analysis_code_and_data folder, together with the Python code for the sensitivity analysis reported in Figure S3 and Table S5 (subfolder cns_sensitivity_python).

Table S1. Per-patient clinical, immunophenotypic and cytogenetic characteristics of the 21 patients (de-identified).

Table S2. Per-patient abundance of the 21 discriminating metabolites (MS-DIAL peak intensity).

Table S3. Metabolite annotation key: ion mode, MS-DIAL alignment identifier, log2 fold change, FDR-adjusted p value, direction of change, number of samples with no detection, and annotation confidence flag.

Table S4. (a,b) Complete annotated feature matrices for both ionisation modes and all 21 patients (92 positive-mode and 44 negative-mode features).

Table S5. (a–d) Sensitivity analysis excluding the three patients whose relapse was confined to the central nervous system: per-metabolite results for the full and reduced cohorts, model statistics, and all 84 leave-three-relapsed-patients-out subsets.

Figure S1. PLS-DA class-label permutation tests for both ionisation modes.

Figure S2. Variable importance in projection (VIP) scores for both ionisation modes.

Figure S3. Sensitivity analysis excluding the three patients whose relapse was confined to the central nervous system.

Figure S1

(A) *Positive ion mode.*

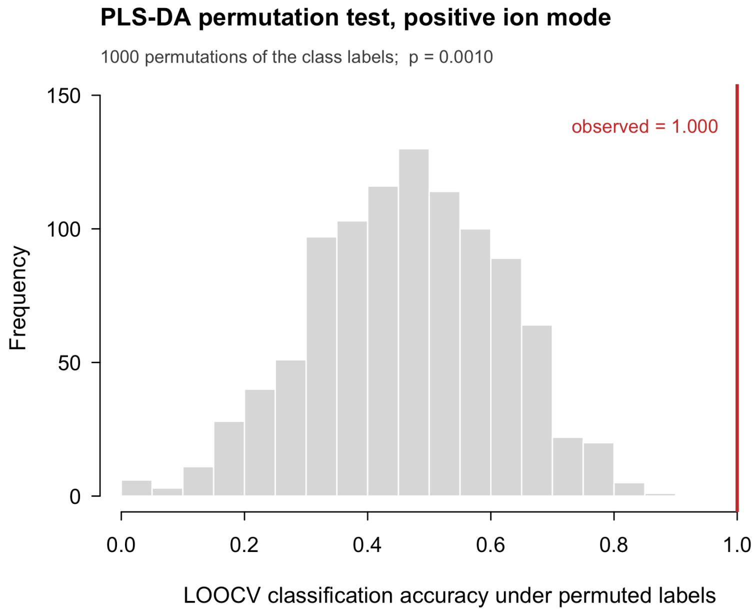

(B) *Negative ion mode.*

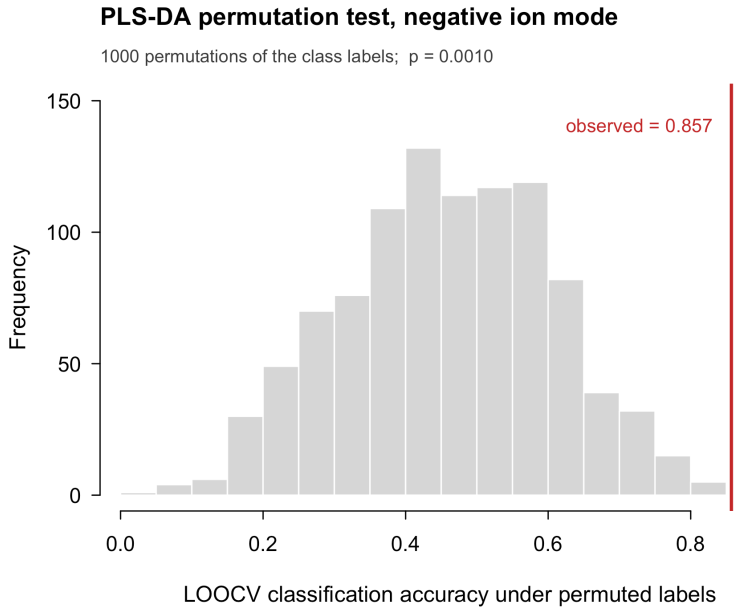

Figure S1. Validation of the PLS-DA models by class-label permutation testing. (A) Positive ion mode. The histogram shows the distribution of leave-one-out cross-validated classification accuracy obtained from 1,000 random permutations of the relapsed and non-relapsed class labels (grey bars; mean accuracy 0.465). The vertical line marks the accuracy of the model fitted to the true labels (1.000, all 21 samples classified correctly), which no permutation equalled or exceeded ($p = 0.001$). **(B)** Negative ion mode. Same analysis; permuted mean accuracy 0.461, observed accuracy 0.857 (18 of 21 samples classified correctly), $p = 0.001$. In both modes the separation achieved by the model exceeds what is obtained by chance, but this describes the 21 samples analysed here and is not a measure of external predictive accuracy.

Figure S2

(A) *Positive ion mode.*

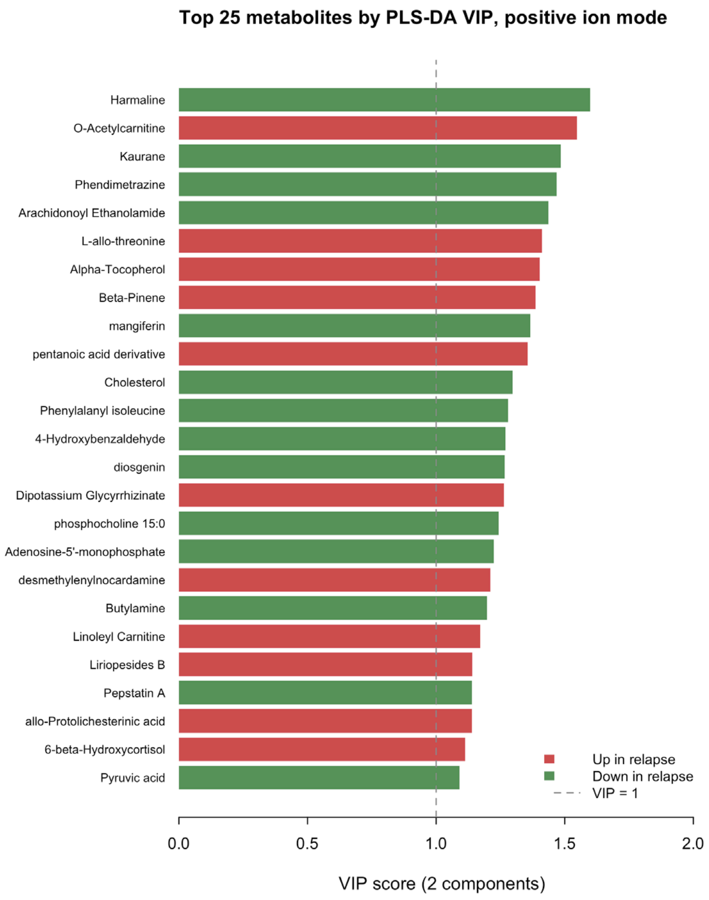

(B) *Negative ion mode.*

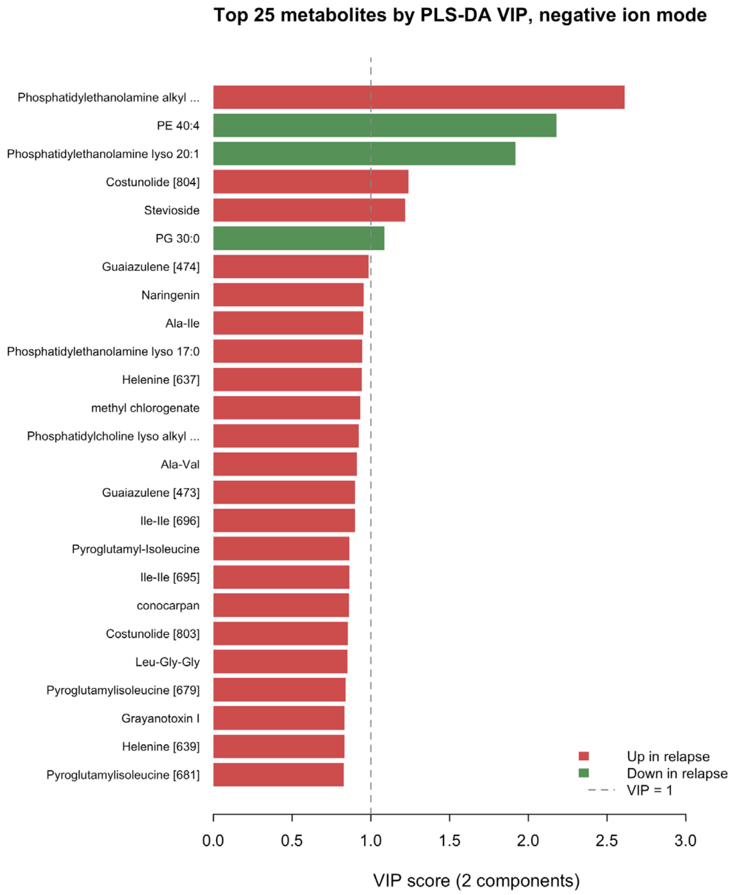

Figure S2. Variable importance in projection (VIP) scores from the two-component PLS-DA models. (A) Positive ion mode. The 25 metabolites with the highest VIP scores are shown as horizontal bars, ordered by score. Bars are coloured by the direction of change in the relapsed group relative to the non-relapsed group: metabolites higher in relapse in one colour and those lower in relapse in the other, as indicated in the panel legend. The dashed vertical line marks VIP = 1, the conventional threshold above which a variable is considered to contribute appreciably to the model. **(B)** Negative ion mode. Same analysis and conventions. VIP scores rank each metabolite's contribution to the multivariate separation and are descriptive; they are not significance tests, and the univariate statistics with Benjamini–Hochberg correction reported in Table S3 should be used to judge which differences are statistically supported.

Figure S3

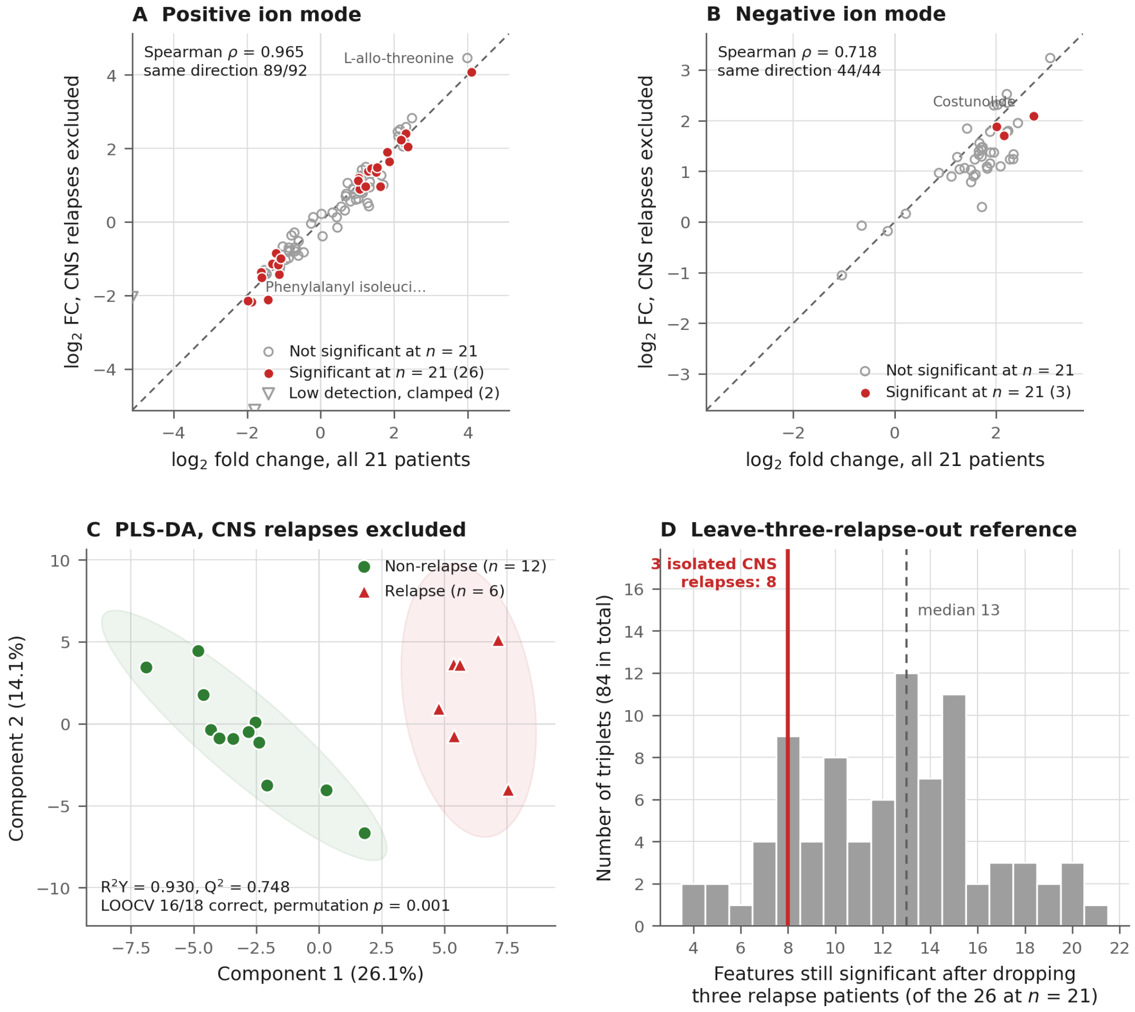

Figure S3. Sensitivity analysis excluding the three patients whose relapse was confined to the central nervous system. (A) Positive ion mode and (B) negative ion mode: the log2 fold change (relapsed versus non-relapsed) computed in the full cohort of 21 patients, plotted against the same quantity computed after excluding the three patients with isolated central nervous system relapse. The dashed line is the line of identity. Red points are the metabolites that were significant in the full cohort (Benjamini-Hochberg $q < 0.05$ and $|\log2 \text{ fold change}| > 1$); the open triangles in (A) are two low-detection features clamped to the axis limits. Spearman correlation coefficients and the number of features retaining the direction of change are annotated on each panel; the coefficient is 0.965 in positive ion mode and 0.718 in negative ion mode. (C) PLS-DA scores for the 18 remaining patients in positive ion mode, with 95% confidence ellipses; model fit, cross-validated predictive ability, leave-one-out classification accuracy and the permutation p value are annotated. (D) Reference distribution for panel (A): the number of the 26 metabolites significant in the full cohort that remain significant after dropping each of the 84 possible sets of three relapsed patients. The dashed line marks the median of that distribution and the solid line marks the subset formed by the three isolated central nervous system relapses, which ranks ninth lowest of the 84.
